# Isolation and Characterization of Neural Stem Cells from the Rat Inferior Colliculus

**DOI:** 10.1155/2019/5831240

**Published:** 2019-10-29

**Authors:** Johannes Völker, Jonas Engert, Christine Völker, Linda Bieniussa, Philipp Schendzielorz, Rudolf Hagen, Kristen Rak

**Affiliations:** Department of Oto-Rhino-Laryngology, Plastic, Aesthetic and Reconstructive Head and Neck Surgery, Comprehensive Hearing Center, Universitätsklinikum Würzburg, Germany

## Abstract

The inferior colliculus (IC) is a nucleus of the auditory pathway and its fourth relay station. It integrates afferent information from the superior olivary complex and the cochlear nucleus. To date, no causal therapeutic options are known for damaged neuronal structures in this area. Regenerative medicine offers a potential approach to causally treating hearing impairment. After neural stem cells had been identified in certain areas of the auditory pathway, the question arouses, whether the IC also has a neurogenic potential. Cells from the IC of postnatal day 6 rats were extracted and cultured as neurospheres. Cells in the neurospheres showed mitotic activity and positive stain of neural stem cell markers (Nestin, DCX, Atoh1, and Sox-2). In addition, single cells were differentiated into neuronal and glial cells shown by the markers *β*-III-tubulin, GFAP, and MBP. In summary, basic stem cell criteria could be detected and characterized in cells isolated from the IC of the rat. These findings will lead to a better understanding of the development of the auditory pathway and may also be relevant for identifying causal therapeutic approaches in the future.

## 1. Introduction

In the course of embryonal development, neurogenesis is responsible for the origin of all forms of neurons in an organism. The embryonic development ends with terminal mitosis, after which the final neuronal daughter cells mature and no further cell division is possible [1]. In recent years, however, postnatal and adult neurogenesis has been identified in two primary zones of the central nervous system: the dentate gyrus of the hippocampus [2] and the subventricular zone (SVZ) [3]. In addition to these primary centers, further neurogenic niches appeared in mammals: the cortex [4], the striatum [5], the septum [5], the spinal cord [6], the dorsal vagal complex [7], and the optic nerve [4]. Since their first description [8], possible functional aspects of neurogenesis for learning processes and memory formation have been discussed [9]. In addition to these characteristics, adult neural stem cells (NSCs) have been of particular interest in regenerative medicine, since they might provide an endogenic healing approach to damaged neuronal tissue and, most importantly, do so without the use of allogeneic embryonic stem cells [10, 11]. The two main characteristics of NSCs are their capacity of self-renewal and their potential to differentiate into neural progenitor cells and into all cells of the neuronal lineage including neurons, astrocytes, and oligodendrocytes [12].

Recently, NSCs were also described in the auditory system. At first, NSCs were detected in the utricle of the vestibular organ [13]. In the area of the cochlea, such a potential was also detected in the spiral ganglion [14] and in the organ of Corti as well as in the stria vascularis [15]. In addition to the peripheral parts of the auditory pathway, stem cell potential was described in the rat [16] and mouse [17] cochlear nucleus (CN). The investigations showed mitogenic properties in cells of this brainstem nucleus in the early postnatal to adult animal with in vitro techniques in cell culture and in whole-mount organ cultures [18]. The formation of neural progenitor cells was proven by specific immunohistological stem cell markers for the intermediate filament Nestin and the microtubule-associated protein doublecortin (DCX). The mitotic capacity of these cells under the influence of mitogenic factors was detected in the BrdU assay and by Ki67, and they also were able to be differentiated terminally into neurons and glial cells. Furthermore, these propagated progenitor cells are able to differentiate and to integrate structurally in the nerve tissue of CN whole-mount explants [19]. Interestingly, the neural stem cells in the cochlear nucleus showed a strong expression of the transcription factor Atoh1, which is an important transcription factor for the derivation of the cochlear nucleus from the rhombic lip [20].

The inferior colliculus (IC) belongs to the central part of the mammalian auditory pathway. It is located in the area of the midbrain, the tectum mesencephali, and, together with the superior colliculi, forms the lamina quadrigemina [21]. The IC consists of three main components: the central nucleus, the dorsal cortex, and the external cortex [22]. The IC, in particular the central nucleus, is beside the cochlear nucleus (CN) and the lateral lemniscus, the fourth relay station of the ascendant auditory pathway, integrating afferent information from the superior olivary complex and the ipsilateral and contralateral CN [23] and thus from the corresponding cochleae. In addition, the IC has input fibers from the auditory cortex, the medial geniculate body, the thalamus, and the superior colliculi. From the IC efferent axons lead to the medial geniculate body and from there further on to the auditory cortical areas [22].

Since neural stem cells have already been discovered in certain regions of the mammalian brain and in the CN of the auditory pathway, the inferior colliculus can also be presumed to have a neurogenic potential. Further analysis of the IC will provide additional information on the development and, possibly, the cell regeneration of this brainstem nucleus. In line with this assumption, the aim of our study was to investigate neural stem cell characteristics *in vitro* and to analyze these cells for their ability to self-renew, proliferate, and differentiate into neurons and neuroglial cells.

## 2. Materials and Methods

### 2.1. Animal and Cell Preparations

Postnatal day (PND) 6 Sprague-Dawley rats (Charles River®) were euthanized by cervical dislocation and decapitation. The skull dome was opened midsagittally, and the bony portions were removed. After incremental raising of the rostral cerebrum, stepwise dissection of the cerebral nerves was performed with microsurgical scissors and the entire brain together with the intact cerebellum and brainstem was removed from the skull base. The brain was immediately transferred into 35 mm Petri dishes (CELLSTAR®, Greiner® Bio-One) in a 5°C DPBS solution (0.05 M, PAA Laboratories®). Using a stereo microscope (ZEISS® Stemi 508), a coronary cut cranially to the lamina quadrigemina was performed in order to separate the cerebrum and brainstem from each other. Under 5x magnification, the blunt dissection of the IC was performed with #5/45 preparation forceps (Dumont®). The preparations were immediately transferred into a sterile DPBS solution (5°C) for further processing. All procedures were performed under antiseptic conditions. All experiments were performed in accordance with the guidelines for animal experimentation under German law (§8, German Animal Protection Act).

### 2.2. Neurosphere Assay, Cell Culture Medium, and Passaging

Following preparation, the neural tissue was transferred to undiluted Accutase (PAA Laboratories®) and dissociated enzymatically in a ThermoMixer (Eppendorf®) at 37°C and 500 rpm for 30 min. Every 10 min, the solution was triturated with a 500 *μ*l pipette until an emulsion of single cells formed in the aqueous solution and no macroscopic tissue parts were visible anymore. After dissociation, cells were centrifuged at 1000 rpm for 5 min (Centrifuge 5810, Eppendorf®) and the resulting pellet was resuspended in stem cell medium (Neurobasal, Thermo Fisher Scientific®). The cell count was determined on a 10 *μ*l sample, which was admixed with 10 *μ*l of trypan blue (Invitrogen®). Thus, the number of vital cells could be counted in an improved Neubauer hemocytometer (ZK06, Hartenstein®) and extrapolated to the total cell count. Free-floating cell culture was carried out in hydrophobic cell culture flasks (CELLSTAR, filter top, 25 cm^2^, Greiner® Bio-One) at 37°C and 5% CO_2_ in serum-free Neurobasal® medium, 1% GlutaMAX® supplement, 2% B27® supplement without retinoic acid, and 1% penicillin/streptomycin (Invitrogen®). The recombinant murine growth factors EGF and bFGF/FGF-2 (PeproTech®) were added to these cultures at a final concentration of 10 ng/ml. This medium is also referred to as NSC medium. At the beginning of the culture time, cells were suspended in 4 ml of medium. Every four days, 2 ml of fresh NSC medium was added. The smallest amount of liquid, which is just enough to cover the entire bottom surface of T25 cell culture flasks, is 4 ml. Therefore, each culture in the experiments started with 4 ml and was then gradually supplied with fresh cell medium. This procedure was possible until d12 of the culture phase when 10 ml total volume was reached. For the addition of fresh medium, a small amount of old medium first had to be removed from the cultures. This was done in an upright position so that the cells sank and were not removed during cell medium aspiration.

The number of primary cell spheres was determined after 30 days with an inverted microscope (Leica® DMI 4000B and DMI-8) at 5x magnification and calculated in relation to 100,000 cultured cells per IC.

For passaging, neurospheres were completely dissociated mechanically. The single cells were again centrifuged for 5 min at 1000 rpm, resuspended in fresh NSC medium, and cultivated at 37°C/5% CO_2_ for 30 days in 50 ml/25 cm^2^ filter top cell culture flasks (CELLSTAR, Greiner® Bio-One). The absolute number of cells in relation to the number of vital cells was determined between each passaging step.

### 2.3. Plating of Spheres

Neurospheres from the free-floating cell cultures were taken with a 5 ml automatic pipette (Multipette plus, Eppendorf®), without mechanically damaging them, and plated on glass coverslips, which had been precoated with poly-D-lysine (100 *μ*g/ml, SERVA Electrophoresis®) and laminin-1 (10 *μ*g/ml, BD Biosciences®). The integrity of neurospheres after pipetting was controlled using a transmitted light microscope (Leica DMI 4000B). In 4-well dishes (Greiner® Bio-One), 100 *μ*l of NSC medium was added per coverslip, and the spheres were cultivated for the intended time for further analysis at 37°C/5% CO_2_. Every two days, NSC medium was replaced by a fresh medium by careful aspiration at the edge of the coverslips with a glass Pasteur pipette and subsequently by the addition of new medium with 100 *μ*l pipettes (Eppendorf®).

### 2.4. Plating of Single Cells and Cell Differentiation

In order to obtain single cells from the propagated cell cultures, the suspension was removed and centrifuged at 1000 rpm for 5 min. The cell pellet was rinsed once with DPBS to remove portions of the cell medium. After suction of the PBS buffer solution, Accutase® was added for cell dissociation and incubated for 15 min at 37°C and 500 rpm in a ThermoMixer (Eppendorf®). Every five minutes, trituration was performed using a 200 *μ*l pipette. Cells were centrifuged, and the pellet was transferred to NSC medium to passage the cultures and to obtain secondary neurospheres. To plate single cells for differentiation experiments, the suspension was transferred into a differentiation medium (DIF medium) consisting of Neurobasal®, GlutaMAX®, and B27 with retinoic acid. Cells were plated on glass coverslips coated with laminin (1 : 100 in 0.05 M PBS) and poly-D-lysine (PDL; 1 : 100 in 0.05 M PBS) at a density of 100 cells/mm^2^ (8000 cells/coverslip) and cultivated in 4-well dishes for eight days at 37°C/5% CO_2_. Medium was changed every two days with DIF medium.

### 2.5. Fixation and Immunocytochemistry

At the end of the individual experiment, cells on glass coverslips were fixed with a 4% paraformaldehyde solution (PFA in 0.1 M NaPP) for 30 min and finally for 5 min with acetone. Blocking of nonspecific binding sites was performed using a solution of 10% bovine serum albumin (BSA, A9418 Sigma-Aldrich®) in 0.1 M PBS buffer solution (Sigma-Aldrich®). For immunocytochemistry, preparations were incubated with the following primary antibodies at 5°C for 12 h in 1% BSA solution and 0.1 M PBS buffer: mouse monoclonal against Atoh1 (1 : 1000; Ab27667, Abcam®), mouse monoclonal against BrdU (5-bromo-2′-deoxyuridine) (1 : 600; #05-633, Millipore®), mouse monoclonal against *β*-tubulin (1 : 1000; #TS293, Sigma-Aldrich®), mouse monoclonal against *β*-III-tubulin (1 : 1000; #Ab7751, Abcam®), rabbit polyclonal against *β*-III-tubulin (1 : 2000; #Ab18207, Abcam®), rabbit polyclonal against doublecortin (DCX) (1 : 1000; #Ab18723, Abcam®), mouse monoclonal against glial fibrillary acidic protein (GFAP) (1 : 1000; #MAB360, Millipore®), rabbit polyclonal against myelin basic protein (MBP) (1 : 800; #M3821, Sigma-Aldrich®), mouse monoclonal against Nestin (1 : 800; #MAB353, Millipore®), and rabbit polyclonal against Sox-2 (1 : 2000; #Ab97959, Abcam®).

After three washing steps in 0.1 M PBS solution, incubation with the secondary antibodies, which were coupled to Alexa Fluor A488 or A555 (1 : 1000; #A11001, #A11008, Thermo Fisher®), was performed with 5 *μ*g/ml DAPI (1 : 5000; D9542, Sigma-Aldrich®) for 1 h in a 1% solution of BSA and 0.1 M PBS. After three final washing steps in 0.1 M PBS solution, the glass coverslips were embedded on object slides with Mowiol® (4-88, Sigma-Aldrich®) and stored at 5°C in the dark.

### 2.6. Analysis of Cell Division

For the analysis of cell division, the number of cells in the specific samples was counted with an improved Neubauer® hemocytometer. Vital cells were determined by adding trypan blue solution (0.4%; #93595, Sigma-Aldrich®) to the measuring chamber. A total of 8000 cells per glass coverslip (78.5 mm^2^; Hartenstein®, coated with poly-D-lysine and laminin-1) were plated in NSC medium with 5-bromo-2′-deoxyuridine (BrdU) (10 *μ*M) and cultivated at 37°C/5% CO_2_. After 48 hours, cells were fixed with PFA (4% solution) and immunohistochemical staining was performed as described.

### 2.7. Cytomorphometric Analysis and Digital Images

Digital images of the cultures and preparations were taken with a Leica® DMI8 fluorescence microscope and Leica Application Suite X software v3.0.1 (Leica®). Tissue sections were analyzed with an Olympus® Fluoview FV3000 confocal laser scanning microscope. To quantify living cells, culture dishes were scanned using a transmitted light technique with a 5x lens in tile scan mode. Subsequent analysis of the acquired image files was carried out with Fiji/ImageJ V2.0.0 software [24]. In order to fully analyze coverslips with immunocytochemically stained preparations, the coverslips were also automatically scanned in the tile scan mode with a 40x lens and subsequently evaluated. The final image composition was done using Adobe® InDesign CC 2018 v14.0 software.

### 2.8. Statistical Analysis

All collected data were compiled using Microsoft® Excel 2018 V16.19 spreadsheets and statistically analyzed with GraphPad® Prism 7.0a software. First, a column analysis (D'Agostino-Pearson omnibus normality test) was performed to determine whether a Gaussian normal distribution of the data was present. Subsequently, data were analyzed using the ordinary one-way ANOVA test followed by the Tukey multiple comparison test. A *p* value < 0.05 was considered to be statistically significant. Reproducible results were obtained from six or more samples.

## 3. Results

### 3.1. Cell Proliferation and Neurosphere-Forming Capacity

**Figure 1:**
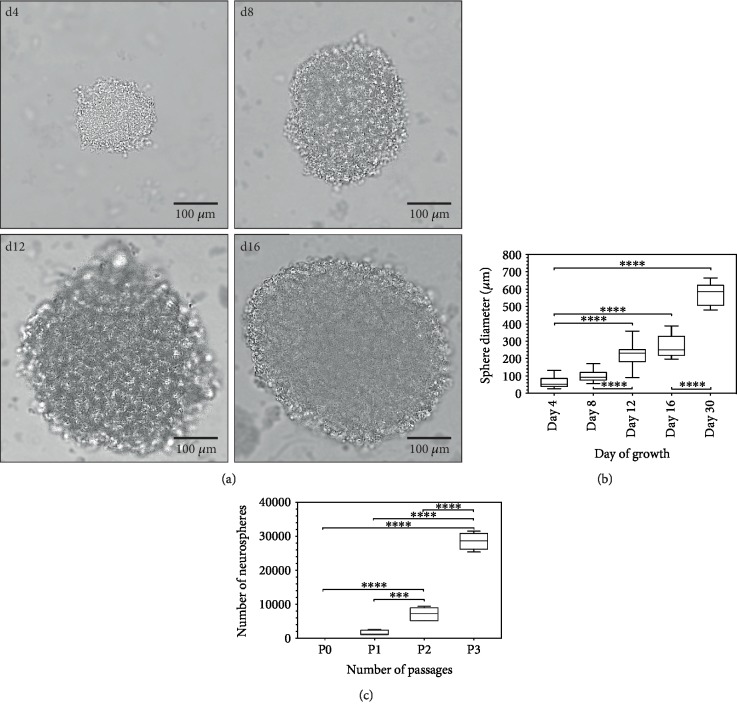
(a) Formation of primary neurospheres from neural stem cells of the postnatal day 6 rat IC in the course of time in free-floating cell cultures with NSC medium containing the growth factors EGF and bFGF (transmitted light microscopy). (b) Primary IC neurosphere diameters with time up to 30 days in NSC medium. There is a significant increase in size from day 4 onwards in culture. (c) Throughout 3 passages for a total of 90 days, the number of spheres at the end of the respective culture period increased significantly. Box plots show the median with the upper and lower quartiles, and whiskers mark the upper and lower maximum values; asterisks indicate the level of significance: ^∗^*p* < 0.05, ^∗∗^*p* < 0.005, ^∗∗∗^*p* < 0.001, and ^∗∗∗∗^*p* < 0.0001.

In free-floating cell cultures of dissociated cells from the IC, spherical cell conglomerates (neurospheres) developed after 4 days. The diameter of these neurospheres increased steadily over time.  Figure 1 shows primary neurospheres of the IC between 4 and 16 days of culture.

Diameters on average increased from d4 (63.82 ± 29.3 *μ*m) to d8 (99.19 ± 29 *μ*m) by 155% (n.s.), from d8 to d12 (226.33 ± 66.6 *μ*m) by 228% (*p* < 0.0001), from d12 to d16 (267.79 ± 60.38 *μ*m) by 118% (*p* < 0.0001), and from d16 to d30 (571.79 ± 59.66 *μ*m) by 214% (*p* < 0.0001). From d4 to d30, this shows an overall increase in size of 896% on average (*p* < 0.0001) (*n* = 30) (Figure 1(b)).

After a period of 30 days, the first passage of the neurospheres was carried out. Secondary neurospheres formed from the isolated cells after the seventh day in NSC medium. Following an additional growth phase of 30 days, tertiary neurospheres could be generated. The total number of cells as well as the number of vital cells in culture continuously increased over time and over the various passages. After 30 days, on average 1588 ± 606 neurospheres per culture/animal or 8.2 ± 3.1 neurospheres per 1000 single cells were determined in primary cell cultures (*n* = 6) (Figure 1(c)). On average, the number of neurospheres increased from P1 (1588 ± 606) to P2 (7170 ± 1752) by 452% (*p* < 0.001) and from P2 to P3 (28524 ± 2125) by 398% (*p* < 0.0001). Overall, there was an increase in the number of spheres by 1796% from P1 to P3 (*p* < 0.0001) (*n* = 6).

**Figure 2:**
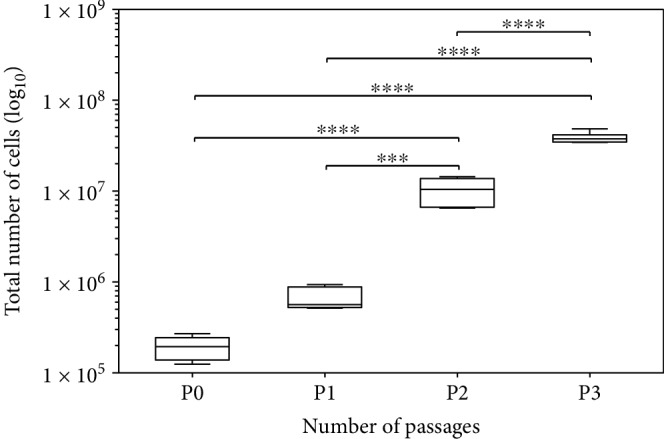
Total number of cells within the neurosphere cultures continuously increased significantly via passages P0-P3. Box plots show the median with the upper and lower quartiles, whiskers mark the upper and lower maximum values, and asterisks indicate the level of significance: ^∗^*p* < 0.05, ^∗∗^*p* < 0.005, ^∗∗∗^*p* < 0.001, and ^∗∗∗∗^*p* < 0.0001.

The single-cell count increased over a period of 30 days (P0 to P1) on average to 13.8 times or 1381% from 193,667 ± 51,037 to 2,674,833 ± 288,221 (*n* = 6) (Figure 2). After complete dissociation of the neurosphere cultures, the trypan blue test showed an average of 24.7% vital cells in P0. Passage from P1 to P2 resulted in an increase in single cells to 10,358,000 ± 3,228,798 (*p* < 0.001), averaging 387%, and from P2 to P3 to 38,723,000 ± 4,747,868 cells (*p* < 0.0001), averaging 374%.

**Figure 3:**
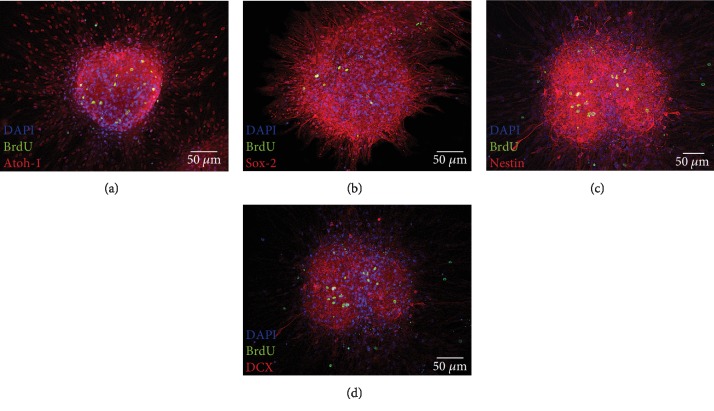
Generation of neural progenitor cells and stem cell division within neurospheres incubated with BrdU (10 *μ*M) in NSC medium for 24 h on glass coverslips. Mitotic cells were indicated by BrdU incorporation (green). Cell nuclei were stained blue by DAPI. Neural progenitor cell markers could be identified and were stained red. Atoh1 was detected in the nucleus, whereas Sox-2, Nestin, and doublecortin (DCX) showed staining in the somata of the cells.

Plated neurospheres adhered after 24 hours on coverslips, coated with poly-D-lysine (PDL) and laminin-1. It was possible to further cultivate these neurospheres over seven days in NSC medium and to analyze them immunocytochemically after fixation. For analysis of cell division within the neurospheres, bromodeoxyuridine (BrdU, 10 *μ*M) was added for 48 hours before fixation. Within the neurospheres, mitotic activity was found, both in the peripheral and in the core regions, which is shown in Figure 3 (green). The quantification of BrdU-stained cells in relation to the footprint of the neurospheres resulted in an average number of 28.15 ± 16.43 BrdU(+) cells per 115,956.56 ± 71,515.39 *μ*m^2^. This results in an average of 27.69 ± 14.78 BrdU(+) cells per 100,000 *μ*m^2^ (0.1 mm^2^).

On PDL and laminin coatings, these neurospheres showed an outgrowth of sphere branches. Single cells showed a tendency to migrate from the neurospheres and to further divide (BrdU-positive staining, green) or to differentiate morphologically. These migrated cells were still in the progenitor cell stage and were labelled positively by different markers. The transcription factor Atoh1 [20], which has been defined as a derivation marker for neural stem cells of the auditory pathway [16] was detected in the cells (Figure 3). In addition, the neural stem cell marker Sox-2 [25] was detected (Figure 3(b)). The progenitor cell marker Nestin also was labelled positively in cells within the neurospheres and their branches (Figures 3(c), red and 4, green). Neuronal migration protein doublecortin (DCX) was likewise detected in the cytoplasm of cells within these spheres (Figures 3(d), red and 4, red). These progenitor cell markers could be detected not only in the *in vitro*-propagated single cells and neurospheres but also under physiological conditions *in vivo* in PND6 rats (Figure 5).

### 3.2. The Capacity of IC Stem Cells to Form Neural Progenitor Cells

Cells were dissociated from neurospheres of the IC and seeded as 2-D cell cultures on glass coverslips for 24 hours in NSC medium. Most of these cells grew in highly branched, dense networks (Figure 6(c)), and only a few remained in a singular state. Individual cells showed morphological signs of differentiation after 24-48 hours. In most cases, multipolar cell branches formed from the cells as shown in Figure 6(a). Individual cells had bipolar morphology (Figure 6(b)). Within 48 hours, mitotic activity was also detectable by BrdU incorporation in these single-cell cultures (Figure 6). Within this period, a quotient of an average of 8.22% BrdU(+) single cells was obtained (1412.8 ± 147 out of a total of 17,180.3 ± 3791) (*n* = 6). After this period, neural progenitor markers were detectable. The transcription factors Atoh1 and Sox-2 were detected within the nuclei. Some of these cells were double-positive for BrdU and the progenitor cell markers (Figures 6(a) and 6(b)). In isolated growing cells, as well as in the neural network, the intermediate filament Nestin was detectable (Figure 6(c)). A double-positive staining of Nestin and the neuronal migration protein doublecortin was shown in these progenitor cells (Figure 6(d)). In 2-D cultures, it was observed that these cells tend to grow in smaller networks (Figure 6(c)). Singular scattered cells were rarely vital for the examination period, and thus, only a small number of them were analyzed after fixation. For a more detailed classification, a systematic analysis of the cell proportions which were positively stained with stem cell markers was carried out. A total of 88,034 cells on glass coverslips were counted and analyzed for their progenitor cell quota. Overall, a share of 3.81 ± 4.5% was positively stained for DCX, 3.11 ± 2.62% for Atoh1, 2.3 ± 3.23% for Sox-2, and 3.33 ± 3.44% for Nestin (Figure 7(a)).

### 3.3. Cell Differentiation and Maturation from Progenitor Cells into Neurons and Glial Cells

For the analysis of cell maturation, the growth factors EGF and bFGF were withdrawn in 2-D single-cell cultures and DIF medium was added. After a few hours, cells in these cultures were also found to adhere to the coverslips, coated with PDL and laminin. After day 6 of culture, cells had undergone morphological differentiation. Neurons with a polarized, bipolar configuration were identified by positive labelling of *β*-III-tubulin (Figure 8(a)). Cells positive for the marker GFAP were multipolar configured with the typical shape of astrocytes. Their branches were in contact with neighbouring cells in neuronal networks (Figure 8(b)). Highly branched, flat-growing cells with large somata and nuclei showed a morphological differentiation into oligodendrocytes. Myelinisation from the distal ends to the proximal region was shown by positive labelling of myelin basic protein (MBP) (Figure 8(c)). A total of 38,940 cells on glass coverslips were counted and analyzed for their percentage of different markers of differentiation. The significantly largest proportion of single cells (47.35 ± 20.01%) (*p* < 0.05) was *β*-III-tubulin-positive and thus indicated a neuronal differentiation. A share of 1.82 ± 1.58% of the analyzed cells was GFAP-positive, and 4.24 ± 3.11% were MBP-positive after a differentiation period of 6 days (Figure 7(b)).

## 4. Discussion

This study proves for the first time the cardinal criteria of neural stem cells within the rat inferior colliculus, the fourth relay station of the ascendant auditory pathway. It was possible to isolate IC NSCs *in vitro* from PND6 Sprague-Dawley rats, to propagate them in free-floating cell cultures over weeks, and to multiply passage them. The cardinal characteristics of neural stem cells—the ability to undergo mitotic division for unlimited self-renewal, the formation of neural progenitor cells, and the multipotency to mature into all cell types of the neuroectodermal line [11, 12, 26]—were demonstrated in this series of experiments.

### 4.1. Stem Cell Microenvironment and Cell Culture System

To carry out the *in vitro* experiments, a cell culture system was used that was specially adapted to the conditions of neural stem and progenitor cells. All studies were carried out in a serum-free cell culture medium (Neurobasal/B27/GlutaMAX) to maintain as much control as possible over the given influencing factors. The impact of specific cytokines in the neural stem cell microenvironment as well as in cell cultures has been previously described in literature [27]. The group of fibroblast growth factors, especially FGF-2 [28, 29], and epidermal growth factors [30] are considered to be of critical importance. These diffusible signals are essential *in vivo* for the embryonic development and patterning of the nervous system [31] as well as for adult neurogenesis [32]. To mimic this microenvironment *in vitro*, these diffusible factors, EGF and FGF-2, were added at a final concentration of 10 ng/ml each to the stem cell medium of the *in vitro* experiments, as previously described in similar studies [3, 33]. In differentiation assays, mitogen-free Neurobasal cell medium with the B27 supplement plus retinoic acid was used. The significance of retinoids *in vitro* for cell differentiation, analogous to an essential role in the maturation of the central nervous system *in vivo*, has already been described [34].

### 4.2. Neurosphere-Forming Capacity

To study the IC on stem cell characteristics *in vitro*, it was dissected microscopically and, after enzymatic dissociation into single cells, exposed to the mitogens of stem cell medium in free-floating cell cultures. The neurosphere assay is a method for studying possible proliferation and self-renewal capacity of neural stem cells, especially if the region of interest could have a silent stem cell niche *in vivo* [5, 11, 12, 35, 36]. Since there are no specific markers of neural stem cells, they have to be identified by indirect methods [12, 26]. In this series of experiments, the ability for unlimited self-renewal and progenitor cell formation was demonstrated. In the course of 30 days, IC cell cultures continuously generated neurospheres, which increased in size. Under these conditions, a quotient of about 1588 ± 606 neurospheres per culture/animal or 8.2 ± 3.1 neurospheres/1000 viable cells (0.8%) was formed. In subsequent experiments after redissociation, cells of these primary neurosphere cultures generated secondary and tertiary neurospheres. The number of neurospheres with respect to the investigated cell number of the IC can be compared with the results from literature. In the area of the cochlear nucleus (CN) of PND6 rats, 1.4 ± 0.4 spheres/1000 cells (0.14 ± 0.04%) could be determined after 3 weeks of culture time [16]. In one study, the mouse CN showed a comparable capacity for sphere formation. However, younger animals (PND3) were examined, which explains the even higher proliferative rate. The anteroventral and posteroventral mouse CN formed about 30 spheres/1000 viable cells, and the dorsal CN about 20 spheres/1000 viable cells [17]. This quotient was higher, for example, in the fourth ventricle of adult mice with approximately 0.2 ± 0.06 neurospheres/1000 viable cells after eight days [37]. On the one hand, this lower quotient could be explained by the different culture conditions, since in that study DMEM/F-12 cell medium was used, whereas in our experiments, only Neurobasal medium was used. The use of various growth factors also has a significant influence on the capacity of neural stem cells to form spheres [38]. Moreover, the differences in the sphere quotient could be explained by the different neurogenic capacities of individual brain regions and also of different species. Previous studies have shown that progenitor cells of embryonic rats have lower growth potential than those of mice [39].

To determine whether the isolated cells of IC not only have a limited potential for mitotic division but also have theoretically unlimited self-renewal and propagation, more passages were generated after the first culture period. In a cycle of 30 days each, three passages of IC neurosphere cultures were created through a series of experiments, which continued to exhibit the property of sphere generation. Quantification of total cell numbers and number of vital cells within the individual passages showed that these also continued to increase over time as a result of an increasing passage number. In this way, cells of the IC exhibit the most cardinal criteria of stem cell potential, since stem cell activity can be detected in the assay by serial passage of neurospheres [40].

### 4.3. Stem Cell Proliferation and Neural Progenitor Cell Formation

In order to study the generated neurospheres for their continuing stem cell properties, they were analyzed immunocytologically. Single spheres from primary, secondary, and tertiary IC cultures were transferred into 2-D cultures and further cultivated in NSC medium. They adhered on PDL- and laminin-coated glass coverslips after 24 h. Spheres formed centrifugally growing branches. Single cells migrated from the neurospheres into the periphery and showed a morphological maturation. In 48 h, individual cells within the neurospheres showed mitotic division, which was detected immunocytochemically by the incorporation of 5′-bromo-2′deoxyuridine (BrdU). This thymidine analog is a reliable tool for identifying mitotic cells and has applications in the identification of neural stem cells [35, 41–43]. This additional information within the IC neurospheres proves that they developed and increased by mitotic division and not by mere cell aggregation.

Different markers were identified not only in cells within the neurospheres but also in cells that moved out of the cell cluster, which indicates their progenitor cell stage. The marker Atoh1 showed nuclear staining. Atoh1 is a transcription factor for the derivation of the auditory midbrain from the rhombic lip [20]. Deletion of Atoh1 leads to a dysfunctional cochlear nucleus. In addition, Atoh1 is one of the main transcription factors in the cochlear development [44].

Furthermore, it is strongly expressed in neural stem cells of the cochlear nucleus [16]. On the other hand, the markers Sox-2, doublecortin, and Nestin stained the somata of the neural stem cells. Sox-2 is a transcription factor in the embryonic development and a classical marker for neural stem cells [20, 25]. Doublecortin (DCX) is a protein known to be a marker of adult neurogenesis and of developing young neurons [45, 46]. Nestin is an intermediate filament protein that is expressed explicitly by neuroepithelial stem cells in the early stages of CNS development [47, 48]. Cryosections of fixed IC were also made for a relation of the results found *in vitro*. In the IC of early postnatal animals, positive staining of the neural progenitor cell markers Nestin and DCX could also be demonstrated (Figure 5).

In further analysis of neurospheres, it is essential to differentiate between dividing neural stem cells and the resulting progenitor cells with limited ability for self-renewal or multipotency [40].

In the IC neurospheres after a BrdU pulse for 48 h, mitotic activity was observed, especially in the center of the spheres. During this period, a number of cells left the cell cluster of the spheres, migrated centrifugally, and also formed branches in a radial direction. These outgrowing cells were usually not stained BrdU-positive, but positive for progenitor cell markers Nestin, DCX, Sox-2, and Atoh1 (Figure 3). It can be concluded that there is a very heterogeneous group of different cells within IC neurospheres: those that are centrally located seem to be individual NSCs, which have a robust mitogenic potential and form further progenitor cells in the nuclear and peripheral regions. Parts of these progenitor cells migrate to the periphery of the spheres and, in 2-D culture, beyond the outer surfaces of the spheres. In these areas, they show signs of the onset of differentiation by morphologically altering and forming cell branches.

Furthermore, IC neurospheres were dissociated, and single cells were plated for immunocytological analysis in NSC medium on glass coverslips and cultured for 24 h. BrdU-positive cells were detected in these 2-D single-cell cultures (Figures 6(a) and 6(b)). Some of these specific cells stained double-positive for the markers Atoh1 and Sox-2. These cells are part of a population of transiently amplifying progenitor cells that typically retain their mitotic activity for a few days in culture [10, 49].

**Figure 4:**
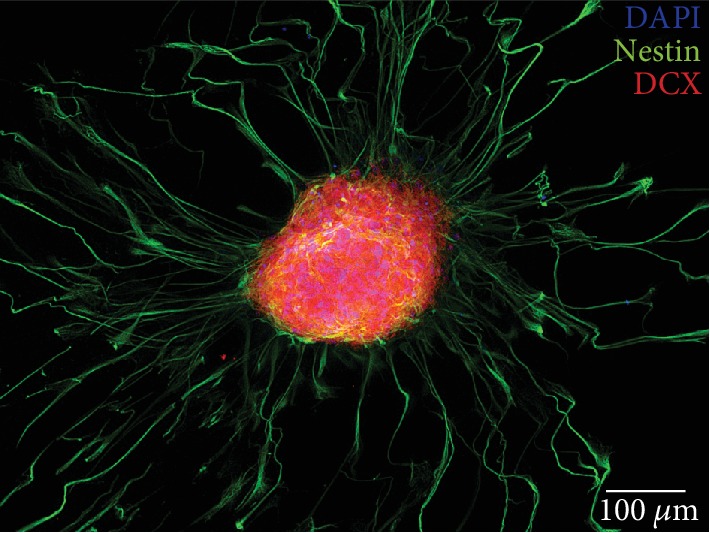
Neurosphere of the IC after 4 days of growth on a glass coverslip. After two days on PDL- and laminin-coated coverslips, these neurospheres showed an outgrowth of sphere branches with an increase in length over the course of time. Nuclei were stained with DAPI (blue). The neural progenitor cell marker Nestin (green) was positive in cells inside the sphere and in the branches. Progenitor cells within the neurospheres were stained positive for the neuronal migration protein doublecortin (DCX) (red).

**Figure 5:**
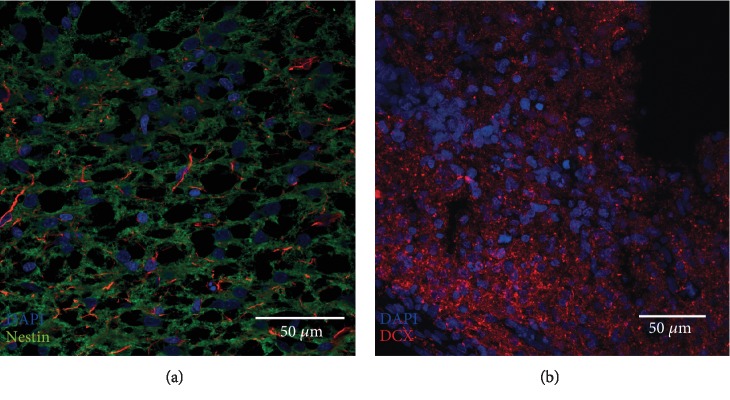
Tissue sections (20 *μ*m) of IC of PND6 rats with immunohistochemical staining of the progenitor cell markers Nestin (a) and doublecortin (DCX) (b).

**Figure 6:**
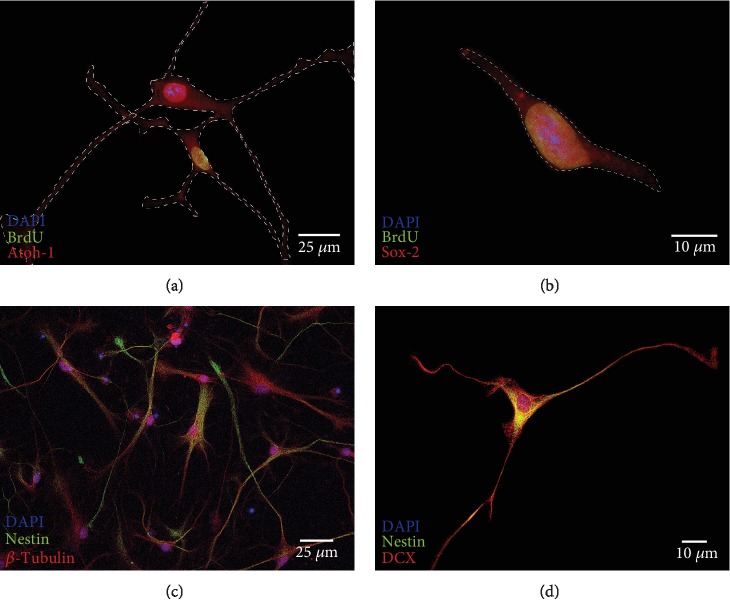
Dissociated cells of neurospheres were plated on glass coverslips and incubated for 24 h in NSC medium. (a, b) BrdU (10 *μ*M) was added to the cell medium. After fixation, immunocytological staining of the BrdU incorporation (green) of single cells and the progenitor cell markers Atoh1 and Sox-2 (red) was carried out. These cells showed positive labelling for the transcription factors Atoh1 and Sox-2, which play a crucial role in the auditory pathway development. (For a better illustration of the somata and cell branches, the contours were drawn with dashed lines.) (c) After incubation in NSC medium for 24 h, cells were fixed. In the network, neural progenitor cells were identified by the marker Nestin (green). Nestin-negative cells were stained by *β*-tubulin and showed the morphology of differentiating bipolar neurons. (d) Neural stem cells of the single-cell culture showed a colocalization of the markers Nestin (green) and DCX (red). Nuclei were stained with DAPI (blue).

**Figure 7:**
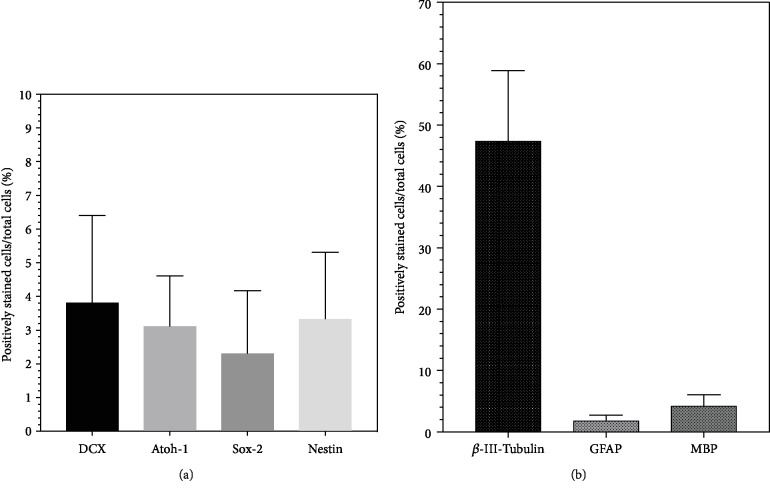
Results of the immunocytochemical analysis of the plated single cells of IC NSC cultures from PND6 rats. The proportions of positively stained cells in relation to the total number of cells on coverslips were evaluated. The boxplots show mean and SEM. (a) Cell proportions that are positive for the analyzed progenitor cell markers after 24 hours on glass coverslips in NSC medium (b) Differentiated cells after 6 days on glass coverslips in DIF medium. Differentiated cell types could be identified morphologically and by immunohistochemical markers of the neuroectodermal lineage. *β*-III-Tubulin stains cells that differentiate into the neuronal lineage, GFAP stains those that differentiate into the astroglial lineage, and MBP stains those that differentiate into the oligodendrocyte lineage.

A key feature of neural stem cells is their asymmetric cell division into progenitor cells and differentiated, nondividing cells [12]. In 2-D single-cell cultures, two different cell types were found after 24 h in NSC medium on the matrix proteins laminin and PDL (Figures 6(c) and 6(d)), a portion of which stained only *β*-tubulin. These cells did not show positive staining for BrdU or progenitor cell markers, indicating their onset of differentiation leaving the progenitor cell stage. Another portion of cells with typical stem cell morphology expressed the intermediate filament Nestin, which is typical of an early progenitor cell stage [48, 50, 51]. This cell type was subsequently observed in a further developed form, which stained doubly positive for Nestin and DCX (Figure 4(d)). This pattern is typical of progenitors with neuronal determination [52].

In summary, it was possible to prove the asymmetric proliferation of cells of the propagated IC neurospheres by typical expression patterns of specific progenitor cell markers and labelling with BrdU after one pulse over 48 h.

### 4.4. Stem Cell Differentiation

**Figure 8:**
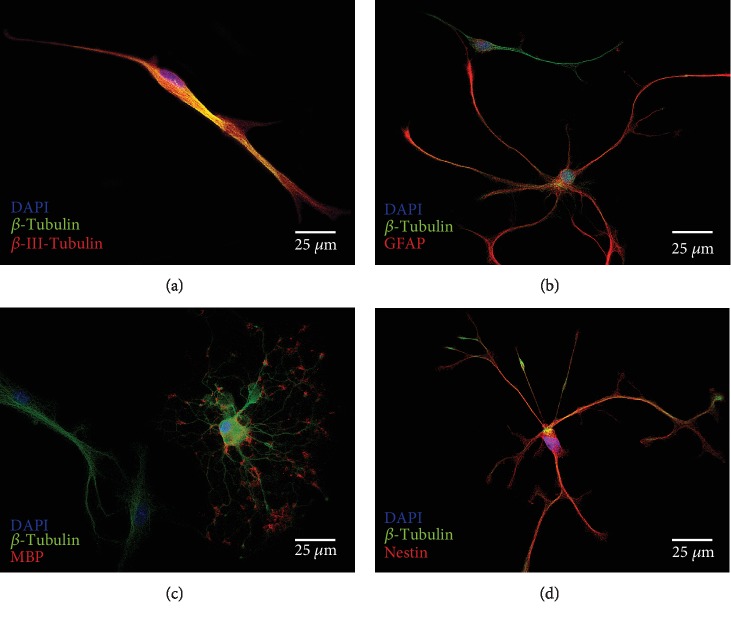
Differentiated cells from IC. NSCs after 6 days on glass coverslips in differentiation medium (DIF). (a) *β*-III-Tubulin (red) marked neuronal differentiated cells. The *β*-III-tubulin-positive cells had neuron-typical spindled, slender somata with bipolar axon configuration. (b) Astrocytes were identified by the staining of glial fibrillary acidic protein (GFAP, red). These cells showed stellate-configured branches, which is typical for astrocytes. (c) Oligodendrocytes showed positive labelling of myelin basic protein (MBP, red). Typically for oligodendrocytes, they had a peripheral-starting myelination, which is characterized by MBP staining. The proximal portions are *β*-tubulin-positive (green). The extensive, diversified growth of the branches is a morphological sign for oligodendrocytes. (d) Cells that were still in the undifferentiated progenitor cell stage were stained by Nestin (red). These cells had a very similar morphology to that of the GFAP-positive, astrocyte-differentiated cells. They have multipolar branches, which are labelled positively for Nestin. The cytoskeleton of all viable cells was stained by *β*-tubulin (green). Cell nuclei were stained with DAPI (blue).

Another essential characteristic of neural stem cells—after transient amplification—is the ability to differentiate into immature neurons and glial cells [47]. Isolated single cells derived from IC neurosphere cultures were able to differentiate into all cell types of the neuroectodermal lineage under withdrawal of growth factors in DIF medium over six days (Figure 8). Within the IC, DIF cultures identified four different cell types: a fraction of multipolar cell branches and roundish somata remained positive for Nestin. These cells were still in a precursor stage and could be identified morphologically and immunocytologically as neural progenitor cells [5, 53]. Differentiated astrocytes were detected by staining with glial fibrillary acidic protein (Figure 8(b)). GFAP is a specific astrocytic intermediate filament marker *in vivo* [54] and *in vitro* [29, 55]. Morphologically, this differentiated cell shape also corresponds to astroglial cells with star-shaped branches, which have been in contact with bipolar neuronal cells. These intercellular contacts serve to supply neurons via connection with blood vessels *in vivo* and play a key role in neurotransmitter regulation and the blood-brain barrier [56, 57]. This type of differentiation has already been described in neural stem cell cultures of the rat's dorsal vagal complex [58]. Bipolar cells with spindle-shaped somata were identified as neurons in these cultures with the marker *β*-III-tubulin. Neuronally differentiated cells formed a network with adjacent, glial differentiated cells (Figures 8(a)–8(c)). These morphological and immunocytological characteristics could also be demonstrated in comparable experiments with NSCs [12, 26]. Within these networks, cells with large somata and spoke-shaped cell branches were found. In the peripheral processes of these glial cells, myelin basic protein (MBP) could be detected immunohistochemically, which identifies them as oligodendrocytes (Figure 8(c)) [59]. These are also adjacent to neuronal cells *in vivo*, where they form the myelin sheaths around axons and play an essential role as a donor of neurotropic substances [60].

## 5. Conclusion

These results support the occurrence of neural stem cells within the IC. In summary, cells isolated from these brainstem nuclei could be propagated in free-floating cultures, where they formed neurospheres. These neurospheres were able to be passaged several times, and by utilizing the BrdU assay, it was possible to prove that the increase in the size of the spheres and the ability to be passaged are due to the capacity to divide and self-renew mitotically. In addition to this essential criterion, it was possible to show the formation of neural progenitor cells both within these neurospheres and from dissociated individual cells using established immunocytochemical markers. If these progenitor cells are further differentiated, the maturation into the primary forms of neuronal and neuroglial cells could be demonstrated. The detection of essential transcription factors that are important for the development of the peripheral and central auditory system provides additional information on the development and possible approaches to the regeneration of the IC *in vivo*. The detection of progenitor cell markers *in vivo* leads to the conclusion that in the postnatal animal, neurogenesis plays an essential role in the development and possibly the regeneration of the auditory pathway.

Since a large number of modulators of neurogenesis, such as growth factors and hormones, have been identified, it may be possible in the future to specifically influence the regeneration of nerve tissue [13]. Possible therapeutic approaches to brain or brainstem damage caused by injuries, toxins, or ischemia could be made possible by a targeted modulation of neurogenesis. In order to be able to regulate neurogenesis therapeutically, however, a detailed understanding of the cellular and molecular processes will be necessary. Specific factors that recruit and propagate neural stem cells are needed, as well as differentiation modulators that enable recruited stem cells to survive and allow them to mature into certain cell lines in future therapeutic approaches.

## Data Availability

The data used to support the findings of this study are included within the article.
